# Clinical Utility of the UPOINT Phenotype System in Chinese Males with Chronic Prostatitis/Chronic Pelvic Pain Syndrome (CP/CPPS): A Prospective Study

**DOI:** 10.1371/journal.pone.0052044

**Published:** 2013-01-17

**Authors:** Zhigang Zhao, Jingwei Zhang, Jun He, Guohua Zeng

**Affiliations:** Department of Urology & Andrology, Minimally Invasive Surgery Center, The First Affiliated Hospital of Guangzhou Medical College, Guangdong Provincial Key Laboratory of Urology, Guangzhou, Guangdong Province, China; Northwestern University, United States of America

## Abstract

**Background:**

Recent data showed that a six-domain UPOINT is a flexible and responsive new classification system that has the clinical applicability in CP/CPPS. However, the utility of UPOINT algorithm in men in China with CP/CPPS has not been comprehensively studied. For international validation and adoption, we evaluated this clinical phenotype system for a large cohort of Chinese CP/CPPS patients and correlated it with patient symptoms and erectile dysfunction (ED). We also investigated the addition of an ED domain in regard to symptom correlation.

**Methods:**

A total of 389 Chinese males with CP/CPPS were prospectively collected and classified in each domain of the UPOINT system. Symptom severity was measured using the NIH-CPSI and IPSS. The erectile function was evaluated using the IIEF-5. Clinically relevant associations were calculated.

**Results:**

The percentage of patients positive for each domain was 54.0%, 42.1%, 41.9%, 20.8%, 26.7%, and 40.4% for the Urinary, Psychosocial, Organ-specific, Infection, Neurological/systemic, and Tenderness, respectively. There were significant correlations between the number of positive UPOINT domains and total NIH-CPSI (r = 0.706, p<0.001), IPSS (r = 0.682, p<0.001) and IIEF-5 scores (r = 0.631, P = 0.007) in Chinese cohort. Except for patients age, symptom duration was associated with a significantly greater number of positive domains (r = 0.638, P  = 0.005). After adding an ED domain to create a modified UPOINT system, the correlation between the number of phenotypic domains and symptom severity was improved (0.706 to 0.844, p<0.001).

**Conclusions:**

The clinical applicability of using UPOINT phenotyping system has been validated in the Chinese patients with CP/CPPS. In our cohort, the number of positive domains was also correlated with ED symptoms and the significant association between the number of UPOINT domains and NIH-CPSI scores was further refined by adding a domain for ED. Our findings presented here support the utility of using ED as a stand-alone item in the UPOINT domain.

## Introduction

Chronic prostatitis/chronic pelvic pain syndrome (CP/CPPS) is a common yet poorly understood condition, with significant economic costs and severe impact on the quality of life of diagnosed patients [1], [2]. The prevalence was estimated between 2.2% and 13.8% [3], [4]. A population-based survey has shown the prevalence of CP/CPPS-like symptoms to be 4.5% in China [5]. Symptoms of this condition include chronic pain, voiding symptoms, and pelvic, sexual and psychosocial disturbances, among others [6]. To date, no single specific therapy is effective in all patients. The major reason for this is that patients with CP/CPPS are not a homogenous group with a single disease process who respond in the same way to specific medications, but rather a heterogeneous group of unique individuals with widely different etiological mechanism(s), disease characteristics, symptom complexes, and progression trajectories. It is therefore rational to evaluate CP/CPPS patients as individuals with differing clinical phenotypes. However, no validated predictors or biomarkers are currently available that help classify those patients in a way that could guide therapy. In 2009, Shoskes et al [7] developed a 6-point clinical phenotyping system called UPOINT to classify patients with CPPS and interstitial cystitis and subsequently direct appropriate therapy. The clinical domains are urinary symptoms, psychosocial dysfunction, organ specific findings, infection, neurological/systemic, and tenderness of muscles. Each domain has been clinically defined, linked to specific mechanisms of symptom production or propagation, and associated with specific therapy. This phenotype is qualitative, with each domain scored as yes or no. The major finding of the first UPOINT retrospective study was the strong correlation between the number of UPOINT positive domains and the NIH-CPSI total score in each patient [8], which was further verified by other studies [9], [10]. More recently, the UPOINT-guided multimodal therapy has been shown to significantly improve symptoms [11].

It is widely acknowledged that CP/CPPS is associated with significant sexual dysfunction [12], [13]. Erectile dysfunction (ED), defined as the consistent inability to obtain and/or maintain a penile erection sufficient for adequate sexual performance, is the most investigated sexual dysfunction in patients with CP/CPPS [12], [13]. The reported ED prevalence findings for CP/CPPS sufferers ranged from 15.0% to 48.3% [14], [15], varying with the evaluation tools and populations. However, the original UPOINT phenotype system did not include a domain for sexual dysfunction or ED. A Swedish study recently showed that the number of positive UPOINT domains was not correlated with ED severity in CP/CPPS patients [9]. The impact of adding a sexual dysfunction or ED domain to the UPOINT system on patients symptoms is also conﬂicting [10], [16].

For international validation and adoption of this novel UPOINT algorithm a prospective study was conducted in a Chinese cohort of males with CP/CPPS. We sought to determine the clinical phenotype of those Chinese CP/CPPS patients using the UPOINT classification system and assessed the frequency of individual domains and their effect on symptom severity and erectile function. Also, we aimed to further investigate the impact of adding an ED domain to create a modified UPOINT phenotyping system on symptom severity of CP/CPPS patients.

## Materials and Methods

### Patients

The patient population included 389 consecutive male patients with a diagnosis of CP/CPPS, who were prospectively evaluated in our Clinic from November 2009 to June 2012, by 1 urologist (Z. Z.). All patients were diagnosed according to the National Institutes of Health (NIH) criteria [17], and assessed by a carefully taken medical history that included a symptom and bother assessment, a focused physical examination that included pre-massage urine and expressed prostatic secretions or post-massage urine analysis and culture, a digital rectal examination (DRE) of the prostate and the pelvic floor muscles, and specifical questions regarding the psychosocial problems. Each patient had their symptom measured using the NIH Chronic Prostatitis Symptom Index (NIH-CPSI) questionnaire, reported as the total score (0–43 points) and the NIH-CPSI subscores for pain (0–21 points), urinary (0–10 points) and quality of life (QoL, 0–12 points) [18], and the international prostate symptom score (IPSS) questionnaire. On the basis of the total NIH-CPSI score, patients were stratified as having mild (0–15 points), moderate (16–29 points), or severe (>29 points) symptoms [8]. The presence and severity of ED was measured for each patient by using the 5-item International Index of Erectile Function (IIEF-5) questionnaire [19]. Any patients with acute or chronic bacterial prostatitis, a history of genitourinary cancer, previous prostate procedures or surgery, and neurologic disease affecting the bladder were excluded from the study.

The patients in this study were a mixture of newly diagnosed, relatively treatment naive (usually antibiotics or anti-inflammatory agents) and tertiary referral patients in whom multiple previous therapies had failed. Prior to commencing this study, the approval from the Ethics Review Board of Guangzhou Medical College was granted, and the written informed consent was obtained from each patient.

### UPOINT Domains

From the clinical data a yes/no classification for each of the six UPOINT domains was made for each individual patient. For each domain the same criteria as repored in the paper by Shoskes et al. were used [8]. In brief, the urinary domain was positive if patients had a NIH-CPSI urinary score >4 and complained of bothersome urgency, frequency, nocturia, or had a postvoid residual urine volume of >100 ml. The psychosocial domain was positive if patients complained of depression, poor coping or catastrophizing, helplessness, hopelessness, or had a poor social interaction. The organ specific domain was considered positive if patients had tenderness localized to the prostate, leukocytosis in the prostatic ﬂuid, hematospermia, or extensive prostatic calcifications. The infection domain was positive if gram-negative *bacilli* or *Enterococcus* was localized to the prostatic ﬂuid in the absence of a current or previous urinary tract infection, or patients had a documented successful response to antimicrobial therapy. The neurological/systemic domain was considered positive if patients had a pain experienced beyond the abdomen and pelvis, with the concurrent diagnoses of irritable bowel syndrome, fibromyalgia, or chronic fatigue syndrome. Finally, the tenderness domain was positive if painful spasms or pelvic floor-related intra-rectal trigger points were detected.

In the present study, we adapted the validated IIEF-5 questionnaire to define patient erectile function phenotype, which was added as an independent ED domain into the UPOINT to create a modified UPOINT system. The ED domain was positive if there was a IIEF-5 score <22. Based on the IIEF-5 score, the severity of ED was divided into five levels, i.e. normal (22–25 points), mild (17–21 points), mild to moderate (12–16 points), moderate (8–11 points), or severe (5–7 points) [19].

### Statistical Analysis

Assuming a two-sided significance test with significance level a = 0.05 for the current study, with our sample size of 389 cases, the estimated power was of 90%. For descriptive statistics, the data are presented as the mean±standard deviation (SD) and medians with interquartile range for continuous variables and counts or frequencies with percentages or proportions for categorical variables. Analysis of variance was used for comparison between multiple groups, and Bonferroni’s multiple comparison test was then used to compare pairs of groups. For comparison of categorical data, the nonparametric Kruskall-Wallis test was used. Correlations of the number of positive domains with symptom severity and ED were evaluated by using the Spearman’s coefficient of rank correlation. Intergroup differences between NIH-CPSI or IIEF-5 scores in patients positive or negative for each domain were analyzed by using the non-paired, non-parametric Mann-Whitney test. Correlation coefficients were also calculated using nonparametric tests. In the study, all analyses were performed with the Statistical Package for the Social Sciences (SPSS) statistical software package version 16.0 for Windows (SPSS, Chicago, IL).

## Results

### Patient Clinical Presentation

Patient age, diagnosis, and the results of the NIH-CPSI, IPSS and IIEF-5 scores and the 6 UPOINT domains in the Chinese cohort are listed in Table 1. The 389 men had a median age of 43 years (range 19–73), with a median duration of symptoms of 9.3 months (range 1–156). The median total NIH-CPSI score was 23.7 (range 6–43), and the median total number of UPOINT positive domains was 3 (range 1–6). The number of patients having positive domains was 21 (5.4%) for 1 domain, 123 (31.6%) for 2 domains, 167 (42.9%) for 3 domains, 51 (13.1%) for 4 domains, 20 (5.1%) for 5 domains, and 7 (1.8%) for 6 domains. No patient had zero positive domains. The majority had positive scores between 2 and 3, and one-fifth had a score ≥4.

**Table 1:** Demographic and clinical characteristics of the patient cohort.

Characteristics	Eligible patients (n = 389)
**Age (y)**	
Mean±SD (range)	44.62±10.27 (19–73)
**Duration of symptoms (month)**	
Mean±SD (range)	11.0±3.3 (1–156)
**CP/CPPS diagnosis, n (%)**	
Inflammatory (Type IIIa)	164 (42.2)
Noninflammatory (Type IIIb)	225 (57.8)
**NIH-CPSI scores, mean**±**sd (range)**	
Total score	24.51±7.02 (6–43)
Pain subscore	10.68±3.17 (0–21)
Voiding subscore	4.55±2.63 (0–10)
QoL subscore	8.42±2.38 (2–12)
**IPSS scores, mean**±**sd (range)**	11.26±7.50 (2–12)
**Symptom severity, n (%)**	
Mild	92 (23.7)
Moderate	209 (53.7)
Severe	88 (22.6)
Median	
**IIEF-5 scores, mean**±**sd (range)**	22.63±5.41
**ED severity, n (%)**	
No ED	268 (68.9)
Mild	29 (7.5)
Mild to Moderate	40 (10.3)
Moderate	34 (8.7)
Severe	18 (4.6)
**Positive UPOINT domains, n (%)**	
Urinary	210 (54.0)
Psychosocial	165 (42.4)
Organ specific	163 (41.9)
Infection	81 (20.8)
Neurological/systemic	104 (26.7)
Skeletal muscle tenderness	157 (40.4)

### Correlation between the UPOINT Phenotyping Domain and Patients Symptoms

To validate the original findings of Shoskes et al [8], we calculated the correlation between the number of positive UPOINT domains and the NIH-CPSI total scores and NIH-CPSI pain, voiding and QoL impact subscores in Chinese cohort of patients with CP/CPPS. Analysis was also extended to the IPSS scores and to the IIEF-5 scores. The results revealed that there was a strong and significant correlation between the number of positive domains and the NIH-CPSI total score (Spearman r = 0.706, p<0.001) and the IPSS score (Spearman r = 0.682, p<0.001). The number of positive UPOINT domains was also strongly correlated with the NIH-CPSI QoL subscore (Spearman r = 0.650, p<0.001) and pain subscore (Spearman r = 0.643, p = 0.004), but not with the urinary subscore (Spearman r = 0.328, p = 0.140). The correlation between age and NIH-CPSI scores was nonsignificant (Spearman r = 0.136, P  = 0.547), however, symptom duration was found to be associated with a significantly greater number of positive domains (Spearman r = 0.638, P  = 0.005).

**Figure 1:**
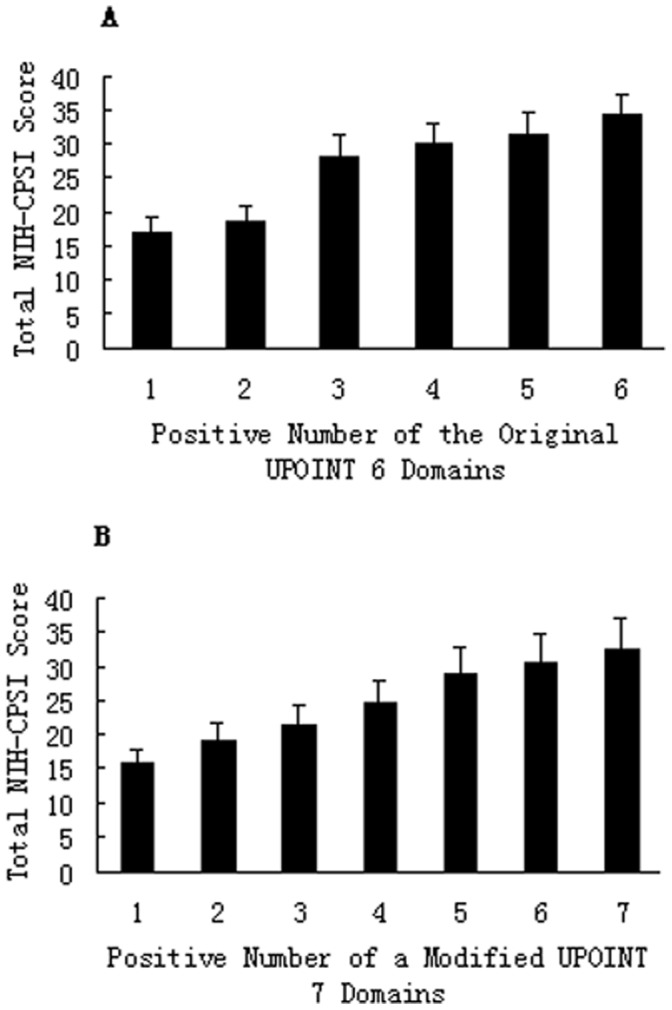
Correlation between NIH-CPSI symptom scores and UPOINT(S) positive number. Mean total NIH-CPSI symptom scores were compared with the positive number of the original UPOINT 6-domains (A) and a modified UPOINTS 7-domains created by adding an ED domain (B), respectively. Significant difference was seen in the NIH-CPSI total score between groups in A and between groups in B (all P<0.001), respectively. Correlation coefficient r improved from 0.706 (A) to 0.844 (B).

**Figure 2:**
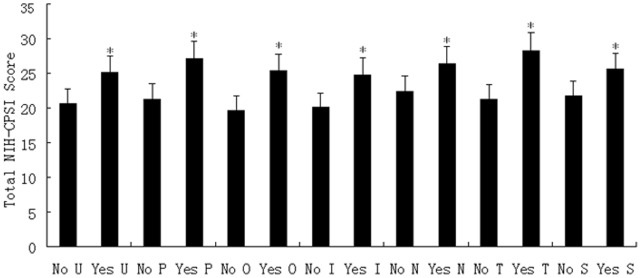
Comparison of NIH-CPSI symptom scores between patients with and without each UPOINTS domain. Mean total NIH-CPSI symptom scores were compared between patients with and without each urinary (U), psychosocial (P), organ specific (O), infection (I), neurological/systemic (N), tenderness (T), and sexual dysfunction (S) UPOINTS domain, respectively. Significant difference was seen in each domain between no and yes (*P<0.05).

As seen in Figure 1A, a stepwise increase was found in the total NIH-CPSI score as the number of positive domains increased from a mean of 16.85±2.11 for patients with 1 positive domain to 34.07±3.16 for those with 6 positive domains (P<0.001, analysis of variance). Stratifying patients by the total NIH-CPSI score as having mild, moderate, or severe symptoms revealed that, as the symptom severity increased, so did the number of positive domains (mild 1.54±0.18, moderate 2.42±0.20, and severe 3.77±0.25; P<0.001, Kruskall-Wallis test). In addition, when the total NIH-CPSI score was compared for the presence of each phenotypic domain, significantly increased symptom scores were seen in all UPOINT domains, including the added sexual dysfunction “S” domain (no *vs.* yes for each domain, all P<0.05, Figure 2).

### Correlation between the UPOINT Phenotyping Domain and ED

**Figure 3:**
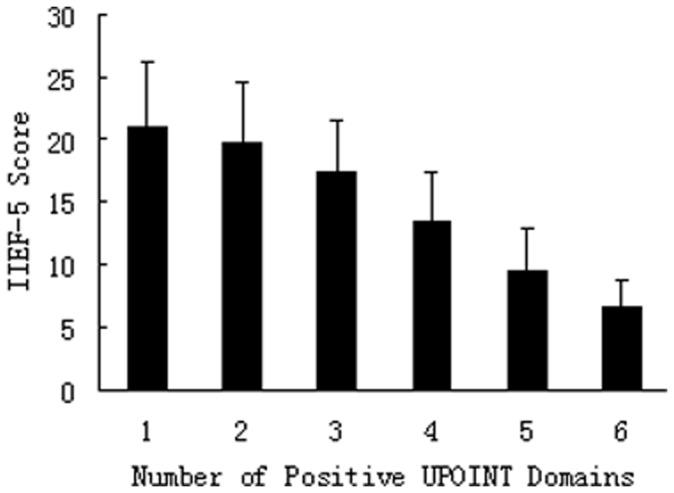
Correlation between IIEF-5 scores and UPOINT positive number. Mean IIEF-5 scores were compared with the positive number of urinary (U), psychosocial (P), organ specific (O), infection (I), neurological/systemic (N), and tenderness (T) UPOINT domains. Significant difference was seen in the IIEF-5 score between groups (all P<0.001), and the number of positive UPOINT domains was significantly inversely correlated with the IIEF-5 scores (Spearman r = 0.631, P = 0.007).

In the study, we also investigated the correlation between the number of positive UPOINT domains and the presence and severity of ED. Our data showed that there was a significant inverse correlation between the number of positive UPOINT domains and IIEF-5 scores (Spearman r = 0.631, P = 0.007). As the number of positive domains increased, the IIEF-5 scores reduced from a mean of 21.04±5.16 for patients with 1 positive domain to 6.59±2.14 for those with 6 positive domains (P<0.001, Kruskall-Wallis test, Figure 3). Stratifying patients by the IIEF-5 score as having no ED, or mild, mild to moderate, moderate, or severe ED revealed that, as the ED severity increased, so did the number of positive domains (normal 0.56±0.15, mild 1.54±0.22, mild to moderate 2.60±0.37, moderate 3.74±0.52, and severe 5.05±0.71; P<0.001, Kruskall-Wallis test).

**Figure 4:**
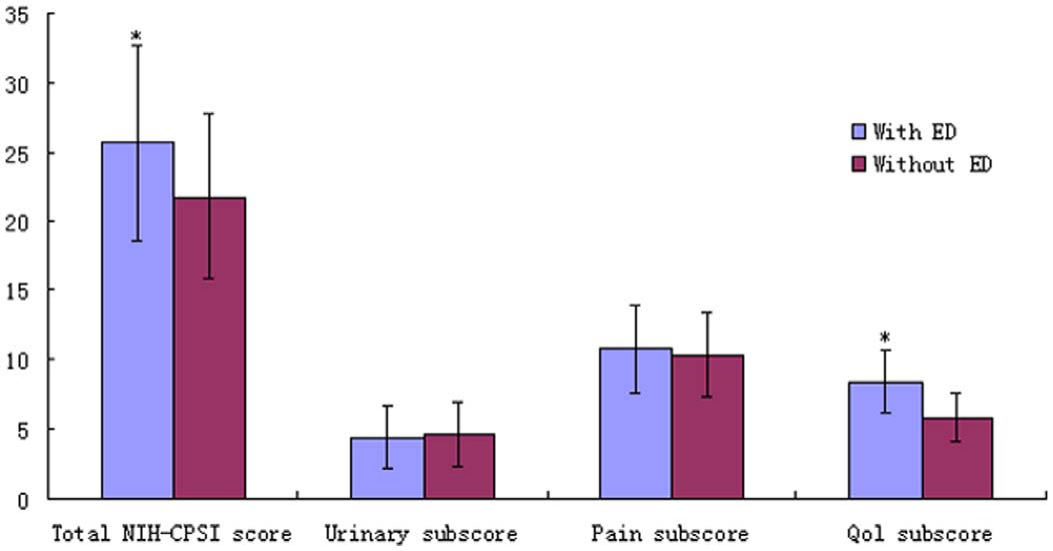
Correlation between NIH-CPSI symptom scores and ED. Total NIH-CPSI symptom score and its urinary, pain and QoL subscores were compared between CP/CPPS patients with and without ED. The presence of ED had a significant effect on the total NIH-CPSI score and its QoL subscore, but not on its urinary and pain subscores (*P<0.05).

**Figure 5:**
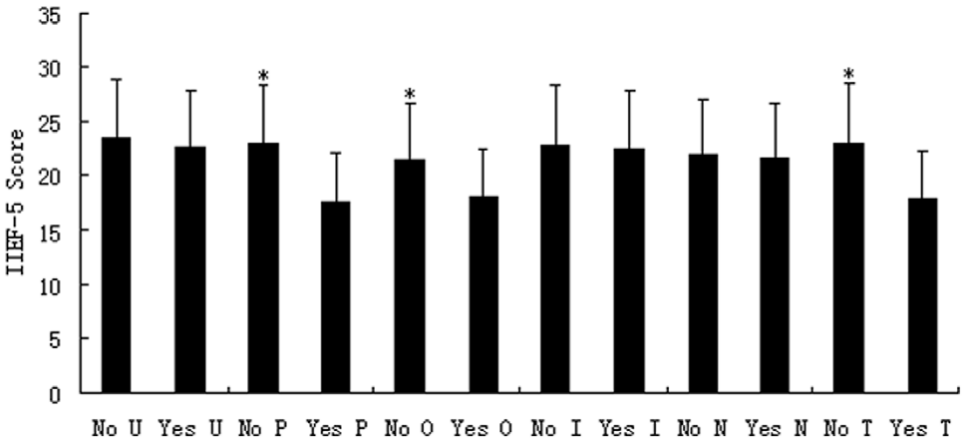
Comparison of IIEF-5 scores between patients with and without each UPOINT domain. Mean IIEF-5 scores were compared between patients with and without each urinary (U), psychosocial (P), organ specific (O), infection (I), neurological/systemic (N), and tenderness (T) UPOINT domain. Significant difference was seen in psychosocial, organ specific, and tenderness domains between no and yes (*P<0.05).

As shown in Figure 4, the presence or absence of ED had a significant effect on the total NIH-CPSI score (25.65±7.10 *vs.* 21.73±5.94, P<0.001) and its QoL subscore (8.41±2.26 *vs.* 5.78±1.79, P<0.001), but not on its urinary (4.38±2.24 *vs.* 4.61±2.32, P = 0.517) and pain subscores (10.75±3.22 *vs.* 10.36±3.05, P = 0.328). When the total IIEF-5 score was compared for the presence of each phenotypic domain of the UPOINT, significantly reduced IIEF-5 scores were seen in patients positive for the psychosocial, organ specific and skeletal muscle tenderness domains (no *vs.* yes for each domain, all P<0.05, Figure 5).

### Effect of Adding a Domain for ED to Create a Modified UPOINT on Symptoms

To evaluate the impact of adding a domain related to ED on symptom severity, we analyzed the correlation between the number of positive phenotypic domains and the NIH-CPSI or IPSS scores in a modified UPOINT system. After adding the ED domain, the correlation between the number of the modified UPOINT domains and the NIH-CPSI total score was significantly improved in our cohort of CP/CPPS patients (correlation coefficient r from 0.706 to 0.844, p<0.001, Figure 1B). Intergroup variance also became significant (Mann-Whitney test, p<0.001). Furthermore, adding an ED phenotype to the original UPOINT significantly improved the correlation between the number of UPOINT domains and the NIH-CPSI pain and QoL subscores (correlation coefficient r from 0.643 to 0.772, p<0.001 and from 0.650 to 0.794, p<0.001, respectively). Similarly, we did not note a significant correlation between the NIH-CPSI voiding subscore and the number of domains of this modified UPOINT domains.

## Discussion

It has become increasingly clear that each patient with CP/CPPS represents a distinct etiological and symptom profile, resulting in a unique clinical phenotype that may be clinically directive, involving clearly identifiable symptom domains to define a heterogeneous population of unique individuals [7], [8]. Classifying patients according to the clinical phenotype will allow for helping determine effective therapy for CP/CPPS [20]. The UPOINT clinical phenotyping system, developed by Shoskes et al [7], has been recently evaluated and validated in the American and European populations of CP/CPPS [8]–[11]. For international validation and adoption of this novel algorithm, we applied the original UPOINT phenotype domains prospectively to a large population of Chinese males consecutively diagnosed with CP/CPPS and evaluated its clinical utility. Our results presented here showed that most patients can be typically categorized with multiple domains and the increasing number of positive UPOINT domains characterizing the clinical phenotype in each patient strongly and significantly correlated with severity of symptoms as measured using the NIH-CPSI or IPSS total scores. The results are consistent with the major findings of the four separate studies that have now examined the UPOINT system in CP/CPPS, in 1469 patients [8]–[11]. Taken together, those data demonstrate that the UPOINT phenotyping system can identify CP/CPPS patients into relevant domains using standard clinical assessment and that the phenotypes yielded by this novel classification system show a heterogeneity for individual domains and the number of positive domains in CP/CPPS. The prevalence and distribution of positive UPOINT domains observed in Chinese patients is remarkably similar to the ones recorded in European or North-American subjects [8]–[11]. In our cohort, the NIH-CPSI values are also strikingly similar to the ones described in the studies of Shoskes et al [8] and Magri et al [10]. These worldwide similarities demonstrate the robustness of both NIH-CPSI and UPOINT systems. Concordantly with the results of the first UPOINT retrospective study [8], we found that the duration of symptoms but not age is significantly associated with an increase in the number of UPOINT domains a patient is identified with, suggesting a progression of multiple domains as the duration of symptoms increased. These results also supported the hypothesis that ongoing local tissue injury and inﬂammation can lead to local muscle spasm, central and peripheral neurologic changes (allodynia, hyperalgesia), and psychosocial changes that can maintain the clinical syndrome years after the initiating injury has resolved [21]. Furthermore, we noted that all six domains of the original UPOINT system did exert a significant effect on both the total NIH-CPSI score and its QoL section subscore in Chinese cohort of patients with CP/CPPS. Thus, it is not surprising that the symptom impact should be more severe if more than one of the domains is involved in the individual patient.

Interestingly and importantly, we found a significant inverse correlation between the number of positive domains and IIEF-5 scores. In our cohort of 389 CP/CPPS patients, as the number of domains increased, the severity of ED measured by the IIEF-5 questionnaire was significantly associated with the number of domains patients experienced. This was not in accordance with the recent finding of Hedelin et al. [9], where no correlation between the number of positive domains and the IIEF-5 scores was observed. This discrepancy may be related to the differences in the sample size, the different criteria used for the UPOINT domains, and the geographic or racial/ethnic dissimilarities. A cohort of 50 patients in the study of Hedelin et al. [9] might be a relatively small-sized sample. In the same study, the criteria used for the UPOINT domains were modified. In their organ specific and infection domains, transrectal ultrasound of the prostate and microscopic examination and cultures of the prostatic fluid were all omitted, that could be considered as a flaw. In the absence of an appropriate LUT segmented test to obtain prostate-specific specimens, a diagnosis of prostatitis is only tentative, and nothing can be said about positivity or negativity of the “I” domain. ED is apparently quite common among men with CP/CPPS seeking medical care, and men with CP/CPPS are more likely to experience ED than age-matched controls [13], [22], [23]. Most recently, a case-control study by using a population-based dataset in Taiwan of China showed that cases with ED were more likely to have had a previous CP/CPPS (odd ratio: 3.62, 95% confidence interval: 3.07–4.26) after adjusting for the patients’ socio-demographic characteristics, comorbidities, obesity, and alcohol abuse/alcohol dependence syndrome status, when compared with controls [22]. In the current study, ED is present in 31.1% of Chinese patients with CP/CPPS. These findings demonstrated a significant association between CP/CPPS and ED in China. As noted by Lee et al. [15], we further observed that ED had an adverse impact on Chinese males with CP/CPPS, who had worse total NIH-CPSI score and QoL subscore than men without ED (p<0.001). Thus, men with CP/CPPS and ED have been shown a tendency to experience substantially worse symptoms, particularly QoL, than other patients with CP/CPPS. In the study, we found that three of the six domains, psychosocial, organ specific and tenderness of skeletal muscle, did exert a significant effect on the IIEF-5 score, suggesting that both organic and extraprostatic factors are all associated with the development of ED in CP/CPPS patients. Although the underlying mechanisms that exist between CP/CPPS and ED remains unclear, many factors can impact erectile functioning, including vascular deficiency, neuromuscular damage, and psychosocial difficulties [24], [25]. It has been suggested that CP/CPPS impairs patients' quality of life and causes mental distress, such as depression, that may be in turn the trigger for ED [26], [27]. Using penile Doppler ultrasonography, Gonen et al [28] demonstrated that none of the men with CP/CPPS and ED showed evidence of vascular deficiency that would interfere with the ability to get an erection, suggesting that psychogenic causes, mainly depression, were the main reasons for the development of ED in CP/CPPS. Several studies have suggested that ED and CP/CPPS may be linked by a shared inﬂammatory process originating from a prostatic source [29]–[31]. It may be possible that such prostatic inﬂammation affects smooth muscle relaxation and impair microvascularization of the prostate [32], thus decreasing the ability of penile tissue to fill with blood and maintain an erection [22]. Also, inflammation of the prostate might impair chemokine, nitric oxide synthase and cyclooxygenase-2 production, and impaired nitric oxide disponibility has been associated with ED [33]. Furthermore, the inflammation and edema of the prostate might impinge on the surrounding neurovascular bundle leading to the onset of ED [34]. Recently, Shoskes and his colleagues demonstrated in a case–control study that men with CP/CPPS were more likely to have abnormalities in their peripheral arterial tone than asymptomatic control patients [35]. The extrinsic compression from pelvic floor spasm could be related to the poor arterial inflow, and therapy with myofascial release can lead to significant improvement in ED symptoms [36].

It appears especially important to detect ED with its deleterious effect on symptoms and quality of life in patients with CP/CPPS. Therefore, we believed that ED merited consideration as a potential unique clinical phenotype domain of CP/CPPS. However, ED or sexual dysfunction was not one of the domains included in the initial UPOINT system. In the present study, when a seventh domain for ED was added into the original UPOINT system, the correlation between the number of this modified UPOINT domains and the NIH-CPSI total score was significantly improved and all 7 domains of the modified UPOINT system showed significantly higher symptom scores. Furthermore, when the ED phenotype was added, patients showing a positive ED domain were characterized by significantly worse NIH-CPSI symptoms. Our results are consistent with those from a large European study by Magri et al. [10], where the 15-item IIEF questionnaire was adapted to define the sexual function phenotype of patients in the frame of a modified “UPOINTS” domain, and the improved correlation occurred not only when the sexual dysfunction domain was defined by the single ED question (16-item questionnaire) but also when orgasmic dysfunction and impaired libido were added to the sexual dysfunction phenotype (17 and 18-item questionnaires). However, our results are inconsistent with those from a recently retrospective study of 100 patients by Samplaski et al. [16], where they did not find improvement in the correlation after adding a seventh domain for ED. The reason for the discrepancy could have had several causes, including patient selection, methodologic differences, and geographic or racial/ethnic dissimilarities. Except for its retrospective nature, a significant limitation of Samplaski’s study [16] was that they did not use a validated questionnaire such as the IIEF to assess for sexual dysfunction. Instead, a yes or no answer to the question “do you have problems achieving or maintaining an erection?” was subjective and insufficient to exhaustively describe the ED profile of a patient. Taken together with the results of Magri et al. [10], those findings suggest that inclusion of the ED or sexual dysfunction domain to the UPOINT phenotype may enable better characterization of the symptom profile in patients with CP/CPPS. Therefore, we propose that an ED domain should be standed-alone in the UPOINT phenotyping system, and currently in our clinical practice we have simulated in our patient cohorts the inclusion of an independent ED item in the UPOINT domains. Additionally, in our cohort a stepwise increase was also found in the NIH-CPSI pain and QoL subscores, but not in the voiding subscore, as the number of positive domains increased. This association hold true when adding an ED domain to create a modified UPOINT system. Absence of correlation with the NIH-CPSI voiding subscore was also shown in the European validation paper by Magri et al. [10]. The poor correlation between voiding symptoms and positive UPOINT domains may be related to the low responsiveness of the NIH-CPSI voiding subscore [37].

A limitation of our study was that we did not account for confounding risk factors for ED. Comorbidites, including diabetes, peripheral vascular disease, and coronary artery disease, can all also contribute to ED, and these were not specifically evaluated in this study. In addition, the fact that the patients were examined and diagnosed by a single physician in the study carries with it a risk for inclusion bias, but it may contribute to the homogeneity of the studied population.

### Conclusions

The utility of the UPOINT classification scheme in discriminating clinical phenotypes has been validated in the Chinese cohort of CP/CPPS, with the number of positive domains shown to strongly correlate not only with the duration and severity of prostatitis symptoms but also with the ED symptoms. Adding an ED domain restored a significant association between the number of a modified UPOINT domains and the NIH-CPSI scores. Although the correlation between ED and the UPOINT domains still needs confirmation in extensive clinical trials, our prospective analyses demonstrate that ED is an important component of the clinical phenotype of CP/CPPS and the addition of an ED domain to the UPOINT system adds a value to the clinical assessment of CP/CPPS symptom severity. The results presented here support the utility of using ED as an independent UPOINT domain.
